# Quality of life among patients receiving palliative care in South Africa and Uganda: a multi-centred study

**DOI:** 10.1186/1477-7525-9-21

**Published:** 2011-04-08

**Authors:** Lucy E Selman, Irene J Higginson, Godfrey Agupio, Natalya Dinat, Julia Downing, Liz Gwyther, Thandi Mashao, Keletso Mmoledi, Tony Moll, Lydia Mpanga Sebuyira, Barbara Ikin, Richard Harding

**Affiliations:** 1 King's College London, Dept. Palliative Care, Policy and Rehabilitation, Cicely Saunders Institute, Bessemer Road, Denmark Hill, London SE5 9PJ, UK; 2 Hospice Africa Uganda, PO Box 7757, Makindye, Kampala, Uganda; 3 The Division of Palliative Care, Department of Internal Medicine, University of the Witwatersrand, Theatre Road, The Chris Hani Baragwanath Hospital, Soweto, South Africa; 4 Formerly of the African Palliative Care Association, PO Box 72518, Plot 850, Dr Gibbons Road, Kampala, Uganda; 5 Hospice Palliative Care Association of South Africa, PO Box 38785, Howard Place, 7450 Suite 11a, Lonsdale Building, Lonsdale Way, Pinelands, 7430, Cape Town, South Africa; 6 Palliative Medicine Unit, Faculty of Health Sciences, University of Cape Town, Observatory 7925, Cape Town, South Africa; 7 Church of Scotland Hospital, P/Bag X502, Tugela Ferry 3010, KwaZulu Natal, South Africa; 8 Infectious Diseases Institute, Faculty of Medicine, Makerere University, PO Box 22418, Kampala, Uganda; 9 Msunduzi Hospice, Head Office, PO Box 22023, Mayors Walk 3208, Pietermaritzburg, KwaZulu Natal, South Africa

## Abstract

**Background:**

Quality of life (QOL) is a core outcome of palliative care, yet in African settings there is a lack of evidence on patients' levels of QOL. We aimed to describe QOL among patients with incurable, progressive disease receiving palliative care in South Africa and Uganda, to compare QOL in cancer and HIV, to determine how domains of QOL correlate with overall QOL, and compare levels of QOL in this population with those in other studies using the same tool.

**Methods:**

A cross-sectional survey was conducted using the Missoula Vitas Quality of Life Index (MVQOLI), a 26-item QOL questionnaire with five subscales (*Function, Symptom, Interpersonal, Well being, Transcendent*) covering physical, social, psychological and spiritual domains and one global QOL item. One item in each subscale assesses the subjective importance of the domain on a score from 1 (least important) to 5 (most important), used to weight the contribution of the subscale towards the *Total QOL *score. The tool was translated into 6 languages and administered to consecutively recruited patients at four facilities in South Africa and one in Uganda.

**Results:**

285 patients were recruited, with a mean age of 40.1; 197 (69.1%) were female. Patients' primary diagnoses were HIV (80.7%), cancer (17.9%) and other conditions (1.4%). The mean global QOL score was 2.81 (possible range 0 (worst) to 5 (best)); mean *Total *score 17.32 (possible range 0 to 30). Patients scored most poorly on *Function *(mean 0.21), followed by *Well being *(2.59), *Symptoms *(5.38), *Transcendent *(5.50), *Interpersonal *(9.53) (possible range for subscale scores -30 to 30). Most important to patients were: close relationships (mean 4.13), feeling at peace (4.12), sense of meaning in life (4.10), being active (3.84), physical comfort (2.58). Cancer patients were predominantly recruited at three of the sites; hence comparison with HIV-infected patients was restricted to these sites. HIV+ patients (n = 115) scored significantly worse than cancer patients (n = 50) on *Well being *(*Z *= -2.778, p = 0.005), *Transcendence *(*Z *= -2.693, p = 0.007) and *Total QOL *(*Z *= -2.564, p = 0.01). Global QOL score was most weakly correlated with *Total QOL *(r = 0.37) and the *Transcendent *subscale was most highly correlated (r = 0.77) (both p < 0.001). Patients receiving palliative care in South Africa and Uganda exhibited significantly poorer QOL compared to similar populations in the USA.

**Conclusions:**

Feeling at peace and having a sense of meaning in life were more important to patients than being active or physical comfort, and spiritual wellbeing correlated most highly with overall QOL. It is therefore vital to identify and meet the psychological and spiritual care needs of patients, as well as to assess and treat pain and other symptoms. Our finding that patients scored most poorly on the *Function *domain warrants further research.

## Introduction

The burden of progressive, life-limiting disease in sub-Saharan Africa is reflected in the epidemiology of HIV [1,2] and cancer [3]. In sub-Saharan Africa in 2007 there were 22.5 million people living with HIV infection; 1.7 million adults and children became infected with HIV; and 1.6 million died of AIDS [1]. In addition, cancer and other non-communicable diseases are becoming urgent public health issues in the region. Parkin et al. report that approximately one in five deaths in sub-Saharan Africa is due to cancer; by 2050, the lifetime risk of cancer is expected to increase by 50-60%, and the annual number of cases to rise from 650, 000 to 2.2 million [4]. The burden of other progressive non-malignant diseases is unknown.

Palliative care is therefore an essential component of public health services in sub-Saharan Africa; however, current provision in the region is patchy, and coverage is poor [5,6]. Successful models of community- and home-based palliative care in the region have been described, but also significant challenges, [5] including lack of access to essential drugs [7], poor social conditions, [8] high morbidity and mortality in health workers, [9] and a lack of trained palliative care professionals [10].

Despite these difficulties, Uganda and South Africa are internationally recognised to have made sustained gains in the provision of palliative care, largely through community-based hospices and home-based palliative care services. Uganda was the first country in Africa to make palliative care for people with HIV and cancer a priority in its National Health Plan (2001-2005) [11]. Oral morphine is available in districts with specialist palliative care clinicians, and nurse prescribing is legislated, [12] although problems with morphine roll-out are documented [13].

In South Africa, progress has involved the inclusion of morphine as an essential drug in primary care, with national standards for cancer pain management [14,15]. The first palliative care services were hospices founded in the 1980s in line with UK models; [14] however, palliative care is now provided through hospitals and home-care providers across the country. South Coast Hospice (one of our participating facilities) has pioneered the successful Integrated Community-based Home Care (ICHC) model, [16] and a national network of hospices operates according to accredited standards. The first fully integrated public sector palliative care service has also been launched in the largest hospital in South Africa [17].

A major barrier to the further development of palliative care in sub-Saharan Africa is the lack of data to inform service provision [10,18]. Palliative care research in Africa has predominantly focussed on opioid availability and physical aspects of care, such as the assessment of pain and other symptoms, while neglecting holistic outcomes such as quality of life (QOL). QOL is defined by the World Health Organization as 'an individual's perception of their position in life, in the context of the culture and value systems in which they live and in relation to their goals, expectations, standards and concerns.' [19] In developed country settings, QOL assessment has become increasingly important as healthcare providers attempt to understand the impact of healthcare interventions on patients' lives rather than solely their physical outcomes [20]. In particular, the main focus of palliative care is to improve the QOL of patients and their families who face the problems associated with life-limiting illness [21]. This includes meeting patients' social, spiritual and psychological needs as well as alleviating pain and other physical symptoms [22]. In the developed world there has been rapid growth in QOL research that aims to understand patient experience, identify patient needs and evaluate the effectiveness of interventions and services [21,23,24]. However, to date there has been very little research in this area in Africa, [5,18] despite recognition of the importance of outcome measurement and the need to identify domains in which patients may need specific support [25,26]. Poverty, HIV stigma and multiple AIDS deaths within the same family are common in sub-Saharan Africa, [27-29] and likely to impact on QOL, hence findings from high income countries are unlikely to be applicable in the African context.

Few studies have examined QOL and non-physical aspects of the experience of serious illness in Africa, with some exceptions. Qualitative research has identified a need for improved communication [30], financial support and good symptom control [31] in sub-Saharan palliative care settings. In HIV infected populations in sub-Saharan Africa, high levels of both physical and psychological symptoms have been reported, [32,33] and significantly lower scores on QOL as measured by the MOS-HIV, than in non-infected populations [34]. However, a limitation of the MOS-HIV is its omission of items relating to spiritual well being, a unique predictor of QOL in other settings [35]. Poor QOL has also been found among patients with HIV in South Africa (N = 607) [36] using the WHOQOL-HIV [37,38]. However, the WHOQOL-HIV is a 100-item questionnaire with an additional 38 importance items, and is reported to take 45-60 minutes to complete [37-40]. This makes the tool overly burdensome for research with unwell palliative care participants [41,42]. In Uganda, a Luganda version of the Missoula Vitas Quality of Life Index (MVQOLI), a 26-item measure originally developed and validated in the USA [43], was administered by Namisango et al to 200 patients with advanced AIDS in urban Kampala [44]. The authors found the poorest QOL in the domains of function, psychological well being and physical symptoms. However, these studies [32-34,44] focus on HIV-infected patients, and levels of QOL in samples that represent the range of clinical and demographic characteristics of patients seen by palliative care services in South Africa and Uganda are not known.

The primary aim of this study was therefore to describe QOL among patients with incurable, progressive disease receiving palliative care in South Africa and Uganda in order to inform clinical care. Secondary aims were to compare QOL in patients with cancer and patients with HIV in our sample, to determine the association between subscale scores and *Total QOL *score, and to compare levels of QOL in this population with the findings of previous studies using the MVQOLI in the USA and Uganda.

## Methods

### Study design

The study we report here is a component of a large, 30-month collaborative project, the Encompass project. During the Encompass project qualitative and quantitative data was collected in four phases during the validation and testing of the APCA African POS [30,45,46]. In the phase reported here, we conducted a cross-sectional survey using the MVQOLI at three non-profit palliative care services and one state service in South Africa and one voluntary sector hospice service in Uganda.

### Participating services

Criteria for selecting the five participating sites were: established palliative care services able to support research and fulfil recruitment criteria for the study, and representing a range of service types (home-based care and inpatient units) and locations (rural, urban township and urban), in order to enhance the generalisability of findings [47]. All services aimed to offer holistic palliative care in line with the WHO definition [22], provided by multi-professional teams that included medical doctors, registered nurses, caregivers/nursing assistants, social workers and (at two sites) counsellors. All services also had access to spiritual care providers (who were paid staff, trained volunteers or community providers). Table 1 shows further details of the services.

**Table 1:** Description of participating palliative care services

Site	Area served	Service type	Source of most of funding	No. of patients cared for (2007-8)	Type of patients cared for	Number of patients recruited (total N = 285)
A	Urban	Home care, day care, outpatient clinic, hospital consultancy, and outreach to healthcare clinics through three branches	Local, national, and international donors	2396	Patients with advanced cancer or advanced HIV	63

B	Urban township	Four sister hospices, all offering short term (2-3 week) inpatient care; two also offer home care	Local, national, international donors	1607	Patients with HIV, cancer, or other progressive incurable conditions	62

C	Urban township	Home care, outpatient clinic, and inpatient unit attached to hospital	Government and international donors	1818	Patients with cancer, HIV, or MND, from diagnosis until point of death	40

D	Rural	Home care and inpatient unit close to hospital	Global Fund for AIDS, Tuberculosis and Malaria	847	Patients with HIV, from diagnosis until point of death	60

E	Rural, peri-urban, and urban township	Home care, day care, and inpatient unit	Local, national, and international donors, including Global Fund for AIDS, Tuberculosis and Malaria	1290	Patients with cancer, HIV, or MND, from diagnosis until point of death	60

MND = motor neurone disease.

### Subjects and recruitment

We included participants in the study if they were adult (at least 18 years old) patients, able to give informed consent, judged to be physically and mentally well enough to participate by their clinical staff, and able to speak either English or one of six local languages fluently (isiXhosa, isiZulu (Gauteng and KwaZulu Natal dialects), SeSotho, SeTswana, Luganda, and Runyoro). The target sample was 60 patients per site, giving a total of approximately 300. This was considered a feasible target given the time available and the size of the services, and a sufficiently large sample size to meet the aims of the study.

We recruited patients consecutively at the five participating services, named sites A-E for the purposes of reporting. A trained researcher was based at each site (GA, TM, KM, and two others - see *Acknowledgements*). At each service, either clinical staff or the researcher approached eligible patients, either on home visits or in the ward. Site A also recruited existing patients by telephone. Service staff or affiliated university departments translated information sheets and consent forms from English into appropriate local languages (see *Data collection*). We obtained informed written consent prior to the interview; illiterate participants were read the information sheet and consent form and marked rather than signed their consent using a thumb print or symbol (e.g. a cross) [48,49]. All patients were informed that refusal to participate would not affect their care in any way.

The study was reviewed and approved by the Ethical Review Boards of the Universities of Cape Town (128/2006), KwaZulu Natal (E025/06) and Witwatersrand (M060366), the Ugandan National Council for Science and Technology (HS143), Hospice Africa Uganda, and the Hospice Palliative Care Association of South Africa (001/06).

### Data collection

#### Instruments

##### Missoula Vitas Quality of Life Index (MVQOLI)

The MVQOLI was developed by Byock and colleagues to measure adaptation to, and integration of, physical and functional decline, as well as attainment of tasks of life completion and life closure in advanced disease [43,50]. The tool contains 26 items: one global QOL item and five subscales, consisting of five items per subscale: *Symptoms, Function, Interpersonal, Well being*, and *Transcendent *(see Table 2 for items). In general, the *Symptoms *and *Function *subscales correspond to the physical domain of patients' illness experience, *Interpersonal *to the social domain, *Well being *to the psychological and *Transcendent *to the spiritual. However, there is some overlap between the psychological and the spiritual, as the *Well being *subscale includes items related to feeling at peace (items 17 and 20), which is arguably a spiritual construct, [51] therefore we considered the *Well being *subscale psycho-spiritual in nature.

**Table 2:** MVQOLI items and subscales

Subscale	Item
**Global QOL**	How would you rate your QOL?

**Symptoms**	1. My symptoms are adequately controlled
	2. I feel sick all the time
	3. I accept my symptoms as a fact of life
	4. I am satisfied with current control of my symptoms
	5. Despite physical discomfort, in general I can enjoy my days *OR *Physical discomfort overshadows any opportunity for enjoyment

**Function**	6. I am still able to attend to most of my personal needs by myself *OR *I am dependent on others for my personal care
	7. I am still able to do many of the things I like to do *OR *I am no longer able to do many of the things I like to do
	8. I am satisfied with my ability to take care of my basic needs
	9. I accept the fact that I cannot do many of the things I used to do *OR *I am disappointed that I cannot do many of the things I used to do
	10. My contentment with life depends upon being active and being independent in my personal care

**Interpersonal**	11. I have recently been able to say important things to the people close to me
	12. I feel closer to others in my life now than I did before my illness *OR *I feel increasingly distant from others in my life
	13. In general, these days I am satisfied with relationships with family and friends
	14. At present, I spend as much time as I want to with family and friends
	15. It is important to me to have close personal relationships

**Well being**	16. My affairs are in order; I could die today with a clear mind *OR *My affairs are not in order; I am worried that many things are unresolved
	17. I feel generally at peace and prepared to leave this life *OR *I am unsettled and unprepared to leave this life
	18. I am more satisfied with myself as a person now than I was before my illness
	19. The longer I am ill, the more I worry about things 'getting out of control' *OR *The longer I am ill, the more comfortable I am with the idea of 'letting go'
	20. It is important to me to be at peace with myself

**Transcendent**	21. I have a greater sense of connection to all things now than I did before my illness *OR *I feel more disconnected from all things now than I did before my illness
	22. I have a better sense of meaning in my life now than I have had in the past *OR *I have less of a sense of meaning in my life now than I have had in the past
	23. As the end of my life approaches, I am comfortable with the thought of my own death *OR *As the end of my life approaches, I am uneasy with the thought of my own death
	24. Life has become more precious to me; every day is a gift *OR *Life has lost all value for me; every day is a burden
	25. It is important to me to feel that my life has meaning

The initial global QOL item is scored from 1 (worse possible) to 5 (best possible). Within each subscale there are two 'assessment' items and two 'satisfaction' items. The final item in each subscale is a subjective measure of that domain's importance to the patient, the importance score; from this score the contribution of the domain to the patients' overall QOL is calculated. In each subscale, assessment items are scored from -2 to +2 and satisfaction items are scored from -4 to +4, in line with the greater role of satisfaction (reflecting mastery and adaptation) in the underlying theoretical construct [43]. The average assessment scores and the average satisfaction scores provide the unweighted dimensional scores, which range from -6 to +6. Weighted dimensional subscores are calculated by multiplying the sum of the average assessment score plus the average satisfaction score by the importance score (an integer between 1 and 5) in that dimension; weighted subscores therefore range from -30 to +30. The *Total QOL *score is calculated by summing the five weighted dimension scores, dividing the score by 10 and then adding 15, so that the resulting total falls between 0 (worst QOL) and 30 (best QOL). The *Total QOL *score therefore reflects the patient's multidimensional QOL weighted according to his/her own identification of the most important dimensions. The MVQOLI had adequate internal consistency (α = 0.77) and broad construct validity (r = 0.43 *Total *score with global QOL) among patients with a prognosis of six months or less at community based hospices in the USA [43].

Although the MVQOLI was not developed for use in Africa, it has been tested in a relevant population in Uganda, one of the countries in which this study was conducted. A slightly modified version of the tool (the MVQOLI-M) was validated in advanced AIDS patients in Uganda [44]. In this study we opted to use translated versions of the original version of the tool for a number of reasons. Firstly, only one of the five study centres was in Uganda and, as South Africa is a more economically developed nation, significant cultural differences were anticipated between the country contexts. Secondly, for the purposes of pooling the data from the five sites a standardised questionnaire was needed; however, the Ugandan version had changed the order of some of the items, so could not be used as a direct equivalent to the original. Thirdly, the Ugandan tool is in Luganda, which was just one of the three Ugandan languages required for data collection in this study. Fourthly, the Ugandan validation made only a few minor changes to the wording of items. Finally, the Ugandan version was validated only in advanced AIDS patients, while our population of interest was all patients seen by palliative care services in South Africa and Uganda, including cancer and other non-communicable diseases.

In order to assess whether or not the MVQOLI was appropriate for measuring QOL in this context, we conducted a factor analysis in a previous publication [52]. Principal component analysis and Varimax rotation were used to determine whether the factor structure of the tool in this study sample replicated the groupings of the original tool dimensions [52]. The factor analysis included all subscale items except the importance item from each subscale, as we found these items behaved differently. A 5-factor solution which closely resembled the subscale structure of the tool accounted for 55% of variance. Internal consistency of the tool using the original scoring method and including all 25 subscale items was α = 0.78 (subscales: *Function *α = 0.52, *Symptoms *α = 0.41, *Interpersonal *α = 0.67, *Well being *α = 0.49, *Transcendent *α = 0.54).

However, the results of the factor analysis [52] suggested minor modification to the domain structure of the MVQOLI may be appropriate in this sample. The structure identified by the factor analysis suggested the following factors: *Interpersonal *(items 11, 12, 13, 14 and 24), *Function *(items 2, 6, 7, and 8), *Well being *(items 16, 17, 18, 19 and 23), *Symptoms *(items 1, 3 and 4) and *Transcendent *(items 21 and 22) (importance items (5, 10, 15, 20, 15) and the global QOL item were omitted from the factor analysis in line with Schwartz et al [53]). We therefore calculated modified subscale scores: the mean score of the items within the subscale, calculated using standardized 'raw' scores, i.e. all items scored on a five point Likert scale (1 = worst possible, 5 = best possible). Internal consistency of the MVQOLI was high, with α = 0.83 for the 20 items scored 1-5 (individual factors: *Interpersonal *α = 0.74, *Function *α = 0.75, *Well being *α = 0.63, *Symptoms *α = 0.56, *Transcendent *α = 0.70). Subject to further testing and confirmation of this factor structure in similar populations, this item grouping may improve the validity of the measure in sub-Saharan contexts.

Following the results of the factor analysis, data are analysed in this study using both the original domain structure and scoring system of the MVQOLI and the modified scoring and factors identified in our factor analysis (see *Analysis*).

##### Demographic data

Research nurses also collected demographic data (see Table 2 for variables). In line with international guidelines, we defined having an AIDS diagnosis as having a CD4+ T-cell count below 200 cells/μ, or having had an AIDS-defining illness, e.g. extrapulmonary tuberculosis. We used the ECOG Performance Status to measure patient functional status [54]. We elected to collect data on the number of children that respondents were responsible for, rather than number of biological children. This was because within Africa multiple AIDS deaths within the same family and a broader understanding of what constitutes "family" mean adults may care for children other than their own, e.g. grandchildren, nephews and nieces.

#### Translation

The participating sites translated the MVQOLI and all other study documentation from English into local languages (isiXhosa, isiZulu [Gauteng and KwaZulu Natal dialects], isiSotho, Luganda, Runyankole and Runyoro). Staff members who were fluent in both English and the relevant local language cross-checked the translations, focussing on conceptual equivalence [55]. The University of KwaZulu Natal conducted the KwaZulu Natal isiZulu translation and the University of Cape Town conducted the isiXhosa translation.

#### Data collection procedure

After receiving training on the administration of the MVQOLI and the demographic questionnaire (LS), independent researchers based at each site administered the questionnaires, either in the participant's home or at the site during a routine outpatient visit or inpatient admission. Patients were informed that participation in the study would not influence the care they received. At site A, two trained local interviewers assisted the researcher, owing to the long distances between the catchment areas of the service's satellite clinics. At site E, one trained local interviewer assisted, as the researcher did not speak isiZulu.

The researchers entered anonymised quantitative data into purpose-designed Excel spreadsheets, subsequently imported into SPSS for analysis by LS. All data was stored securely in locked filing cabinets or password-protected files to ensure confidentiality. Data collection and entry was overseen and checked by Principal Investigators at the participating sites (ND, LG, LMS, TM, BI).

### Analysis

#### Descriptive statistics

We described patients' demographic profiles and MVQOLI scores using descriptive statistics (frequency, mean, standard deviation and median). In order to compare importance scores across the subscales, we conducted unpaired t-tests using mean scores, standard deviations and sample sizes.

#### Comparison of sub-groups

As almost all cancer patients (n = 50) were recruited at sites A, B and C, with 27%, 32.3% and 32.5% of cancer patients recruited at each respectively, we compared this cancer sample to HIV patients recruited at the same services to minimise the risk of differences between the sites confounding the findings. We used non-parametric Mann-Whitney *U *tests to compare scores on the MVQOLI subscales by diagnosis (cancer vs. HIV), as the Kolmogorov-Smirnov test (with Lilliefors approximation) demonstrated that the distribution of scores was skewed for all subscales, and we treated the groups as independent samples given our attempt to minimise site effects. We hypothesised a difference between HIV and cancer groups based on the literature [56,57].

#### Associations between subscales and Total score

In order to determine the relationship between subscale scores and *Total QOL *score we used the Spearman's correlation to correlate mean subscale scores with mean *Total QOL *score. We hypothesised moderate correlations between subscale scores and total QOL score in line with the original validation of the MVQOLI [43].

#### Comparison to other studies

In order to compare our findings to those of previous studies, we conducted unpaired t-tests comparing our MVQOLI data to that reported in other studies, [43,44,58] for mean subscale, *Total QOL *and global QOL scores, using standard deviations and sample sizes.

Throughout the analyses we use the original scoring system for the MVQOLI in order to ensure data are comparable with other studies utilising the tool. In describing the population we also report modified subscale scores based on the modified factors identified in our previous factor analysis (see *Instruments*) [52]. The modified subscale scores are not weighted according to the importance of that domain to patients and are not comparable to the scores reported in previous studies.

All analyses were conducted in SPSS v17 except unpaired t-tests, which were conducted using GraphPad software.

## Results

### Sample characteristics

Over a six week period, we recruited 285 patients across the sites. None of the patients approached declined to take part. Interviews were in seven languages: isiZulu (N = 143, 50.2%), isiXhosa (N = 41, 14.4%), English (N = 41, 14.4%), isiSotho (N = 18, 6.3%), Runyoro (N = 15, 5.3%), Runyankole (N = 14, 4.9%) and Luganda (N = 13, 4.6%).

Table 3 shows participants' demographic characteristics. 80.7% of patients had a primary diagnosis of HIV and 17.9% a primary diagnosis of cancer; 15.2% of HIV infected patients also had cancer. The most frequent cancer diagnoses were cervical cancer (N = 32, 37.2%), Kaposi's Sarcoma (N = 8, 9.3%) and prostate cancer (N = 7, 8.1%), reflecting the epidemiology of cancer in the region and common malignancies in HIV [59]. Participants reported 17 different first languages; the most prevalent of these were isiZulu (N = 140, 49.1%), isiXhosa (N = 43, 15.1%), Runyoro (N = 20, 7.0%), Luganda (N = 20, 7.0%) and Runyankole (N = 18, 6.3%).

**Table 3:** Demographic characteristics

Demographic characteristic	Patients (N = 285)
**Age**	
***Mean (SD)***	40.1 (12.8)
***Range***	18-88

**Gender**	
***Female***	197 (69.1%)

**Responsible for children?**	
***Yes***	232 (81.4%)
***Mean no. children (SD)***	3.1 (2.0) 1-12
***Range***	

**Household size**	
***Mean (SD)***	5.3 (2.5)ª
***Range***	1-14ª

**Location of home**	
***Urban***	53 (18.6%)
***Peri-urban***	53 (18.6%)
***Rural***	179 (62.8%)

**Primary diagnosis**	

***MND***	1 (0.4%)

***Korsakoff's syndrome***	1 (0.4%)

***Multiple Sclerosis***	1 (0.4%)

***Systemic lupus erythematosus***	1 (0.4%)

***Cancer only***	51 (17.9%)

***HIV***	230 (80.7%)

***Of HIV+ pts: *On ART**	127 (55.2%)^b^

**Prior AIDS diagnosis**	192 (83.5%)

**Dual HIV-cancer diagnosis**	35 (15.2%)

**ECOG Functional status**	
***Fully active***	21 (7.4%)
***Restricted***	65 (22.8%)
***Ambulatory***	79 (27.7%)
***Limited self care***	87 (30.5%)
***Completely disabled***	33 (11.6%)

**Primary place of palliative care**	
***Home***	180 (63.2%)
***Inpatient***	74 (26%)
***Outpatient***	13 (4.6%)
***Day care***	18 (6.3%)

**Weeks under palliative care**	
***Mean (SD)***	46.0 (74.8)
***Median***	12.0
***Range***	0-468

ª 3 missing

^b ^1 missing

Consideration of the 5th item of each MVQOLI subscale, which assesses the subjective importance of the domain in patients' lives, shows that close relationships (item 15) were most important across the sample (mean 4.13, SD 0.69), closely followed by feeling at peace (item 20) (4.12, SD 0.69) and having a sense of meaning in life (item 25) (4.10, SD 0.65), followed by being active (item 10) (3.84, SD 0.92), and physical comfort (item 5) (2.58, SD 1.12). These items correspond to the *Interpersonal, Wellbeing, Transcendent, Function *and *Symptoms *domains respectively. None of the differences between scores were significant at p < 0.05.

Comparison of the mean scores on the MVQOLI subscales shows that patients scored most poorly (i.e. with least positive contribution to QOL) on the *Function *subscale, followed by *Well being, Symptoms, Transcendent *and *Interpersonal *subscales (Table 4). However, relatively high mean subscale scores may mask a significant proportion of patients scoring poorly on individual items. For example, 165 patients (57.9%) agreed/agreed strongly with the statement 'The longer I am ill, the more I worry about things 'getting out of control'' (item 19); 115 (40.4%) patients agreed/agreed strongly with 'I feel more disconnected from all things now than I did before my illness' (item 21); and 78 (30.9%) agreed/agreed strongly with 'I have less of a sense of meaning in my life now than I have had in the past' (item 22).

**Table 4:** MVQOLI scores overall and comparison by diagnosis (Mann-Whitney *U *test)

MVQOLI score	Overall mean (SD) (N = 285)	Cancer mean (SD) (N = 50)	HIV mean (SD) (N = 115)	P	*Z*
**Global QOL**	2.81 (1.04)	2.94 (0.98)	2.90 (0.95)	0.78	-0.279

**Symptom subscale**	5.38 (7.32)	7.68 (7.73)	5.88 (8.49)	0.49	-0.694

**Function subscale**	0.21 (11.62)	1.04 (10.68)	-1.10 (11.71)	0.96	-0.051

**Interpersonal subscale**	9.53 (12.78)^a^	14.52 (11.15)	11.18 (11.91) ^a^	0.07	-1.846

**Well being subscale**	2.59 (12.12)	3.95 (12.17)	-1.75 (12.69)	0.005*	-2.778

**Transcendent subscale**	5.50 (12.03) ^c^	8.79 (13.37) ^b^	5.57 (11.23) ^d^	0.007*	-2.693

**Total score**	17.32 (3.70) ^e^	18.44 (3.64) ^b^	16.94 (3.61) ^c^	0.01*	-2.564

* Significant at p < 0.05

^a ^Missing n = 2

^b ^Missing n = 1

^c ^Missing n = 8

^d ^Missing = 7

^e ^Missing n = 9

Scores for the modified MVQOLI factors based on [52] are presented in Table 5. *Symptom *and *Interpersonal *scores on the modified factors were relatively high, while Function score was low. Modified subscale scores for the other two domains lie between these values. As weighting items are removed in the modified subscales, these scores do not reflect the subjective importance of the domain to patients' QOL, with consequent variation in the relative ranking of the original and modified mean subscale scores.

**Table 5:** Scores for modified MVQOLI factors (based on [52], N = 285)

Modified MVQOLI subscale score*	Items	Mean (SD)	Median	Interquartile range	Respondent range
**Modified Symptom subscale**	1, 3, 4	3.69 (0.71)	4.0	3.33-4.00	1-5

**Modified Function subscale**	2, 6, 7, 8	2.83 (0.91)	2.75	2.00-3.50	1-4.75

**Modified Interpersonal subscale**	11, 12, 13, 14, 24	3.62 (0.78)^a^	3.80	2.60-3.80^a^	1.2-5^a^

**Modified Well being subscale**	16, 17, 18, 19, 23	3.18 (0.81)^b^	3.20	2.60-3.80^b^	1-5^b^

**Modified Transcendent subscale**	21, 22	3.25 (1.05)^a^	3.50	2.00-4.00^a^	1-5^a^

* Mean score for included items (raw scores used for all items: 1 (worst possible) - 5 (best possible))

^a ^Missing n = 2

^b ^Missing n = 7

### Comparison of sub-groups

Comparison of scores by diagnosis showed that patients with cancer at sites A, B and C scored significantly higher (i.e. better) than patients with HIV infection at those sites on *Wellbeing *(*Z *= -2.778, p = 0.005) and *Transcendent *(*Z *= -2.693, p = 0.007) subscales, and on *Total QOL *score (*Z *= -2.564, p = 0.01) (Table 4).

### Associations between subscales and Total score

Correlations between subscale scores and *Total QOL *were as follows: *Transcendent *(r = 0.768); *Well being *(0.719), *Interpersonal *(0.661), *Function *(0.604) and *Symptoms *(0.382). The global QOL score was most weakly correlated with *Total QOL *(r = 0.365). All correlations were significant (p < 0.001).

### Comparison to other studies

Table 6 compares the subscale, *Total *and global QOL scores from this study with those from other studies using the MVQOLI [43,44,58]. The American studies involved advanced cancer [58] and mixed advanced cancer and organ failure populations, [43] while the Ugandan study surveyed advanced AIDS patients using a modified but comparable version of the MVQOLI [44].

**Table 6:** Comparison with MVQOLI subscale scores from other studies

Study	Sample (N)		Symptoms	Function	Interpersonal	Well being	Transcendent	Total	Global QOL
This study	Palliative care patients, South Africa & Uganda (N = 285 unless stated)	Mean (SD)	5.38 (7.32)	0.21 (11.62)	9.53 (12.78)	2.59 (12.12)	5.50 (12.03)	17.32 (3.70)	2.81 (1.04)

Namisango et al 2007 [44]	Advanced AIDS patients receiving HIV care from community/home/outpatient clinic, Uganda (N = 200)	Mean (SD)	*1.70 *(9.83)	-*2.05 *(10.81)	**17.44 **(13.03)	*-1.39 *(12.90)	**16.08 **(12.99)	*16.27 *(3.38)	2.68 (0.95)
	
		T (p)	4.72 (<0.001)	2.17 (0.03)	6.65 (<0.001)	3.47 (<0.001)	9.16 (<0.001)	3.17 (0.002)	1.40 (0.16)

Byock & Merriman 1998 [43]	Community-based hospice patients, 68% advanced cancer, 11% end-stage lung disease, 8% end-stage heart disease, USA (N = 173)	Mean (SD)	6.19 (7.32)	**6.09 **(15.27)	**17.64 **(10.92)	5.01 (14.45)	**14.10 **(13.00)	**19.91 **(3.97)	**3.39 **(1.07)
		T (p)	1.15 (0.25)	4.65 (<0.001)	6.94 (<0.001)	1.92 (0.06)	7.15 (<0.001)	7.02 (<0.001)	5.72 (<0.001)

Steele et al 2005 [58]	Home hospice patients, 92.6% advanced cancer, 3.9% HIV, 3.1% COPD, 0.4% other, USA (N = 129)	Mean (SD)	*3.16 *(7.87)	1.72 (15.91)	**14.01 **(11.11)	4.29 (14.46)	**15.55 **(11.61)	Not reported	Not reported
		T (p)	2.79 (0.006)	1.09 (0.28)	3.43 (<0.001)	1.24 (0.21)	7.92 (<0.001)		

Note: Mean scores which are significantly higher than in our study are in bold; mean scores which are significantly lower are italicised.

## Discussion

The mean global QOL score among patients in South Africa and Uganda who were recruited into this study was 2.81 out of a maximum of 5, and the mean *Total QOL *score was 17.32 (possible range 0 to 30). Overall, patients in this study exhibited relatively poor QOL in comparison to studies using the MVQOLI in the USA (Table 6). In particular, mean *Interpersonal *and *Transcendent *scores were significantly lower in our sample than in all previous studies using the MVQOLI both in the USA [43,58] and Uganda, [44] indicating worse QOL in these domains. Our data go against the findings of a qualitative study in Kenya which found that patients' psychological, social and spiritual needs were met by family members, the community and faith groups, [31] and suggest that this is not always the case. The mean *Transcendent *score in our study (5.50) is especially low given the next lowest mean score found on this subscale was 14.10, reported by Steele et al in their study of home hospice cancer patients in the USA [58].

However, overall, patients in our study reported significantly better scores on the *Symptom *subscale than AIDS patients in Uganda and home hospice patients' in Steele et al's USA study [58]. Patients in our study also had significantly better functional and psycho-spiritual wellbeing than AIDS patients in the Ugandan study [44]. This may relate to the physical and psychological burden of living with AIDS and the fact that the patients in the Ugandan study were not receiving palliative care.

As in the Ugandan study, we found that the poorest QOL was reported in the function domain, followed by psycho-spiritual wellbeing (as measured by the Well being subscale), then physical symptoms. These domains were also the worst three in the US studies; however, in a different order: Byock and Merriman report poorest QOL in function, symptoms, and then psycho-spiritual well being; Steele et al report poorest QOL in psycho-spiritual well being, function, and then symptoms. However, in our study scores in the spiritual domain (measured by the *Transcendent *subscale) were also notably low, while in the other studies this was not the case [43,44,58].

Despite the pain and symptoms associated with incurable, progressive disease, physical comfort and being active were judged by participants in this study to be less important to QOL than close relationships, feeling at peace and having a sense of meaning in life, as shown by the relatively low scores on importance items 5 and 10, although these differences were not statistically significant. This is also reflected in the results of the correlation, which show that the *Transcendent, Wellbeing *and *Interpersonal *subscales are most highly correlated with *Total QOL*, with spiritual and psycho-spiritual domains reaching levels of strong correlation (r > 0.7). Our findings support other studies suggesting that physical symptoms and function are not as important to patients with life-limiting illness as other domains of QOL [60-63]. This finding could be due to the acceptance of discomfort and physical limitation and the re-evaluation of goals that can occur through 'response shift' [64,65]. There is evidence that cancer patients tend to readjust expectations to fit their current health and functional status, [66] and that patients in palliative care shift their values away from self-enhancement (e.g. power) and towards self-transcendence (e.g. benevolence) [67]. These findings may reflect coping processes in the face of the uncertainty of living with an incurable, progressive disease [68].

Owing to the lower scores on importance items 5 and 10, the symptoms and function domains were accorded less weight in calculating the *Total QOL *score than the non-physical domains. However, despite this the *Symptoms *and *Function *subscales showed poor mean scores. This suggests that even if response shift or adaptation is evident in the relative importance allocated to the five domains, patients in this study nevertheless experienced considerable suffering and reduction of their QOL owing to uncontrolled or unacceptable symptoms and physical function.

Close relationships, feeling at peace and having a sense of meaning in life were rated as highly important by patients (mean 4.13, 4.13 and 4.10 out of 5 respectively). This supports the findings of other studies regarding the importance of interpersonal and spiritual domains in incurable progressive disease [35,61,69-71].

Our findings also suggest that patients with HIV receiving palliative care in South Africa and Uganda may experience poorer QOL than patients with cancer. HIV-infected patients scored significantly worse than cancer patients on *Well being, Transcendence *and *Total QOL*. In the USA, a study by Sherman et al found that patients with AIDS had lower total QOL scores, and lower psychological QOL than patients with advanced cancer; however, patients with AIDS had higher physical QOL scores [72]. In a study of 2,864 HIV-infected adults in the USA, Hays et al found that patients with AIDS had significantly worse physical functioning and emotional well being than patients with prostate cancer [57]. Our findings also support those of other studies in sub-Saharan Africa documenting the psychological and spiritual burden of living with HIV [32] and the need for support in these areas [73-75].

Modified subscale scores based on factor analysis in this sample and reported elsewhere [52] suggest that symptom control/acceptance of symptoms and interpersonal well being are relatively good, while physical function and acceptance of physical limitations is poor. Unlike the subscales in the original tool, the modified subscale scores are not weighted according to the subjective importance of the domains, and this should be noted when interpreting the modified scores. For example, although overall patients rate highest on symptoms and interpersonal well being with respect to the modified scores, once the weighting aspect is taken into consideration the beneficial impact of symptom control/acceptance of symptoms falls: interpersonal well being, rather than symptoms, has a more positive effect on QOL when the original tool scoring system is used.

Finally, it is interesting that global QOL as assessed by item 1 of the MVQOLI ('*How would you rate your overall QOL?*') was poorly correlated with *Total QOL *(r = 0.37). A relatively low correlation between *Total QOL *and global QOL (r = 0.43) was also reported in the original validation of the tool in the USA [46], although this was higher (0.58) in the Ugandan study of advanced AIDS patients [44]. In our study setting the poor correlation between global QOL and *Total QOL *could suggest that the *Total QOL *score measures a construct different to QOL as understood by study participants. One reason may be that the tool does not assess socio-economic factors which might be of particularly relevance to patients in a resource-constrained setting. Hunger and stigma, for example, are prevalent in this population [44,45] and could adversely affect QOL in a way not assessed by the MVQOLI in its current format.

### Limitations

Our study may have overestimated QOL in patients receiving palliative care in sub-Saharan Africa. South Africa and Uganda, where this study was conducted, are widely recognised as the two African countries with the most advanced provision of palliative care [6]. The services involved were not selected randomly; hence findings may not be generalisable across palliative care services in the two countries. Participating sites were some of the most well-established palliative care services in South Africa and Uganda; patients in this study are therefore likely to be receiving relatively high quality palliative care and may have higher QOL than patients at other services. In addition, patients were required to be well enough to participate in self-report data collection, which may bias our data against those with significant disease progression and nearing the end of life, who may have poorer QOL than patients in this sample. As our findings relate to patients receiving palliative care, it is worth noting that other people with life-limiting illness in sub-Saharan Africa have less access to care than this sample, and hence are likely to have poorer QOL [5]. Further studies are required in other African settings.

Owing to resource constraints we were not able to use the best practice methods of double data entry and tool adaptation involving multiple translations, synthesis of translations, back translation, expert committee review and pretesting prior to psychometric testing of the MVQOLI [76]. Two academic departments carried out two of the translations. To minimise inaccuracies, all translations were cross-checked by bilingual palliative care staff and difficulties in translation were resolved through discussion with the local research team. While the factor analysis suggested the tool might be psychometrically appropriate for measuring QOL in this context, the translations utilised in this study would benefit from further investigation and cognitive testing in the future. The interpretation of our findings is therefore subject to modification on the basis of further testing of the MVQOLI in South African and Ugandan populations in the future.

Another limitation relates to the small cancer sample (n = 50) used when comparing MVQOLI scores in cancer and HIV diagnoses. As data were collected as part of a larger study focussing on psychometrics, no power calculation was used to determine sample size for this survey; hence our comparison of QOL domains by diagnosis should be interpreted cautiously. However, our comparison showed highly significant differences between the HIV and cancer samples for three out of six variables, despite the limited sample size.

The heterogeneity of the services and the populations they care for also affects the generalisability of findings. For example, at site D patients were recruited relatively close to admission (mean 2.9 weeks, SD 5.1), while patients at site E had been under palliative care for a mean of 122.3 weeks (SD 100.6). To take account of these variations we limited our comparison of cancer and HIV sub-groups to those services where the majority of cancer patients were recruited. However, there are likely to be demographic differences between patients in the two groups (e.g. gender, age) which we were not able to take into account in our analyses, hence our comparative findings should be interpreted with caution.

Finally, we used parametric t-tests to check for the significance of differences between our results and those of other studies (Table 6), despite a lack of normality in the MVQOLI distributions in our study. However, this was the only comparison possible given the data reported in other studies.

### Clinical implications

Our findings suggest that patients receiving palliative care in South Africa and Uganda have poor QOL as measured by the MVQOLI. While physical function was the domain with the least positive impact on QOL in this study (*Function *subscale mean 0.21), psycho-spiritual well being had the next least positive contribution (*Wellbeing *subscale mean 2.59). These findings show that it is vital to identify and meet the psychological and spiritual care needs of patients, as well as to rigorously assess and treat pain and other symptoms, and support patients in coping with their functional limitations. This requires multi-dimensional palliative care provided by a multi-professional team. Palliative care providers in sub-Saharan Africa are directed to local guidance on supportive and palliative care [77], spiritual care [78], and counselling [79].

The significantly worse scores on *Symptoms, Interpersonal *and *Transcendent *subscales among HIV infected patients compared to cancer patients support other studies reporting high levels of physical and psychosocial symptoms [32,80,81] and spiritual concerns [82] in HIV.

Population level findings should be interpreted with caution clinically, as we found that relatively high mean subscale scores may mask a significant proportion of patients scoring poorly on individual items. Individual levels of QOL depend on a host of contextual factors, such as patient history, family and community relationships, and spiritual beliefs. The heterogeneity of patient experiences should therefore be taken into account by ensuring that multi-dimensional needs are assessed regularly and that care is patient-centred and tailored to individual patient and family needs.

### Future research

On the basis of our findings we recommend the following areas for future research. First, research is needed to explore the QOL of patients receiving palliative care in sub-Saharan Africa in different settings and using different models of care. Research focussed on specific settings and population groups would enable demographic and clinical predictors of QOL to be identified. Second, our findings support the need for research into psychological and spiritual aspects of patients' illness experience, as these areas are highly important to patients, are rated relatively poorly as domains of QOL, and are currently neglected in palliative care research in Africa. Third, our finding that physical function as measured by the MVQOLI has a significant negative effect on patients' QOL warrants further research. For example, it would be useful to explore current occupational therapy, counselling and home care provision in sub-Saharan Africa and evaluate the effect of these interventions on the functional domain of patients' QOL. Finally, future studies are needed to examine and if necessary improve the translations of the MVQOLI used in this study, explore the comprehension of specific translations through cognitive interviewing, and conduct further testing of the tool's validity, reliability and appropriateness, including assessment of weighting and scoring systems and exploration of factor loadings in populations from different African countries.

## Conclusions

Patients receiving palliative care in South Africa and Uganda have poor QOL as measured by the MVQOLI, and in comparison to hospice patients in the USA. Patients with HIV infection in this population may be at increased risk of poor QOL when compared to patients with a primary diagnosis of cancer, particularly with respect to psychological and spiritual domains.

In this study, close relationships, feeling at peace and having a sense of meaning in life were overall more important to patients than being active or physical comfort, indicating the particular relevance of family, friends and spiritual concerns in this patient group. This supports the need for QOL scales to include psychosocial and spiritual items and not to neglect these aspects of patient experience by focussing solely on physical symptoms. It is vital to identify and meet the psychological and spiritual care needs of patients, as well as to assess and manage pain and other symptoms and functional limitations in this population. QOL is a key outcome of palliative care and should be assessed in future research evaluating interventions and models of care in Africa, in order to inform the provision of palliative care in the region.

## Competing interests

The authors declare that they have no competing interests.

## Authors' contributions

RH and IJH won funding. RH, IJH, and LS designed the study. LS and RH trained the nurses and oversaw data collection. GA, TM, and KM recruited participants and conducted, transcribed, and translated interviews. ND, LG, TM, LS, JD and BI supervised translation, recruitment, data collection and data entry. LS analysed the data and wrote the first draft of the paper. IJH and RH provided support in overseeing the analysis and revision of the drafts. All authors commented on and contributed to the final draft. RH is the guarantor.
